# Problems and Challenges with Analysis of Short Tandem Repeats (STRs) in Animal DNA

**DOI:** 10.3390/ani16152321

**Published:** 2026-07-28

**Authors:** Aleksandra Figura, Magdalena Gryzinska

**Affiliations:** Institute of Biological Basis of Animal Production, University of Life Sciences in Lublin, 20-950 Lublin, Poland

**Keywords:** animal forensics, PCR artefacts, short tandem repeats, genetic identification

## Abstract

The number of households with pets has been steadily increasing in recent years, highlighting the growing social and economic importance of companion animals. The increasing role of animals in human life and the need for biodiversity protection have contributed to growing interest in animal DNA analysis, although it still remains less developed than human forensic genetics. Current research focuses on genetic identification, parentage verification, biodiversity monitoring, and wildlife forensic investigations. DNA identification methods have evolved from restriction fragment length polymorphism (RFLP) to short tandem repeat (STR) analysis, which is currently one of the most widely used approaches due to its high sensitivity, discriminatory power, and the possibility of multiplex amplification. Recent advances include the implementation of massively parallel sequencing (MPS/NGS), sequence-based STR typing, and integrated SNPSTR systems, which significantly improve the interpretation of complex and degraded samples. Despite challenges associated with PCR artefacts, stutter formation, allelic imbalance, and interpretation of low-template DNA, STR markers remain valuable tools in forensic analyses, conservation genetics, and combating illegal wildlife trade. Continued development of new marker panels, standardized nomenclature systems, and bioinformatic approaches is essential for improving the effectiveness and reliability of animal genetic identification.

## Simple Summary

Animals can provide important biological evidence in criminal investigations, breeding programmes, and wildlife conservation. Hair, blood, saliva, and other biological samples can be used to identify individual animals in much the same way as human DNA is used in forensic science. This review explains how short repeated regions of DNA are used for animal identification and describes their applications in forensic investigations, parentage testing, conservation, and the fight against illegal wildlife trade. It also discusses the main challenges associated with analyzing poor-quality or degraded biological samples, interpreting genetic results, and achieving consistent testing methods between laboratories. Recent technological advances and future directions in animal genetic identification are also presented. The information summarized in this review highlights the importance of reliable genetic identification methods and may contribute to improving their application in forensic science, animal breeding, and wildlife conservation.

## 1. Introduction

In recent years, the number of households with pets has been steadily increasing, and approximately 50% of European households declare ownership of at least one pet. According to FEDIAF reports, there were more than 312 million pets in European households in 2021; this number increased to 347 million in 2022 and to 352 million in 2023 [1,2,3]. The newest FEDIAF report published in 2025 indicates that about 139 million European households own at least one pet, corresponding to nearly 299 million companion animals in Europe [4]. These data confirm the continuing growth in the importance of companion animals in European society and the increasing social and economic significance of the pet sector.

Alongside this trend, public awareness of animal welfare has increased substantially, while companion animals are increasingly regarded as family members [5]. Interest in verifying the origin of animals, maintaining accurate pedigree records, and detecting illegal breeding or sales practices has grown considerably. Despite advances in molecular biology and the availability of analytical methods routinely used in human genetics, animal genetic identification remains less developed than its human counterpart [6]. Although animal forensic genetics is increasingly recognized, discussions at forensic genetics meetings and scientific literature remain predominantly focused on human DNA analyses. It was once believed that analysis of non-human DNA did not require such rigorous regulations and standards, and for this reason, such analyses were carried out mainly in academia. Animal DNA analysis is now increasingly recognized as a valuable component of forensic investigations [5]. In addition to the activities of the International Society for Forensic Genetics (ISFG), the International Society for Animal Genetics (ISAG) plays a key role in the standardization of animal genetic testing. Through its Animal Forensic Genetics Committee and species-specific working groups, ISAG develops recommendations and comparison tests supporting individual identification, parentage verification, species determination, and quality assurance in animal forensic genetics [7].

Dogs and cats remain the most common companion animals, and interest in purebred animal breeding continues to grow. Consequently, parentage verification has become an important component of modern breeding programmes and is routinely applied in animal genetics. Genetic analyses help identify pedigree errors resulting from accidental mismatings or intentional fraud, such as double mating or incorrect assignment of offspring to a litter. Beyond parentage verification, genetic testing is widely used to diagnose hereditary diseases, identify traits of breeding importance, and determine coat colour and other phenotypic characteristics [8,9].

## 2. Genetic Individual Identification Methods

Attempts to develop reliable genetic methods for individual identification have been undertaken for numerous domestic and wild animal species. The introduction of DNA-based analyses has substantially improved the accuracy of individual identification and parentage verification, providing valuable tools for breeding programmes, conservation genetics, and forensic investigations [5,10,11]. Forensic genetics has evolved from restriction fragment length polymorphism (RFLP) and variable number tandem repeats (VNTRs) to short tandem repeats (STRs), single-nucleotide polymorphisms (SNPs), mitochondrial DNA analysis, and insertion–deletion (InDel) markers [10,12]. These marker systems differ in their analytical performance, applications, and limitations. Mitochondrial DNA is particularly useful when highly degraded samples are analyzed because of its high copy number per cell, whereas SNPs and InDels can be amplified using short amplicons and are therefore suitable for degraded DNA. STR markers, however, remain the most widely used tool for animal individual identification because of their high polymorphism, strong discriminatory power, and suitability for multiplex PCR analysis [5,13,14]. Earlier marker systems, such as RFLP and VNTR, were not widely adopted for animal individual identification. Their application was limited by the small amount of DNA that can typically be recovered from animal traces, the lack of single-locus VNTR probes for many animal species, and the absence of population databases required for statistical evaluation of genetic evidence [15]. In addition, the lack of suitable comparative material may further limit the applicability of these methods [16].

## 3. STRs (Short Tandem Repeats)

STR analysis is widely used in forensic investigations because animal biological traces recovered from crime scenes are often degraded and available only in small quantities [13]. The material may be degraded and highly fragmented, but this does not reduce the usefulness of the trace, because STRs are short DNA fragments consisting of tandemly repeated motifs of 2–12 base pairs and typically ranging from 50 to 500 base pairs in length [17,18]. Typing of autosomal and allosomal STR markers using fluorescence-based detection is now routinely performed in DNA laboratories worldwide. Reduced-size STR markers, known as miniSTRs, have been developed to improve the recovery of genetic information from severely degraded DNA samples [19]. In addition to the classification of STRs according to the length of the repeat unit, they are categorized as simple, compound, or complex based on the repeat pattern [20]. Initially, markers consisting of dinucleotide repeats were used in DNA analysis, but later tri-, tetra-, penta-, and even hexanucleotide repeats were analyzed [12,20]. STRs in eukaryotic cells are mainly present in non-coding regions. Their occurrence and variability between individuals are due to the crossing-over process in prophase I of meiosis, the mechanism of retrotransposition, and DNA polymerase slippage during replication, considered the main mechanism of STR polymorphism. DNA polymerase slippage involves the incorrect addition of nucleotides that are not present in the template strand, thereby changing the number of repeating units, which becomes fixed during subsequent replication rounds. It should be noted that the frequency of polymerase slippage does not directly correspond to the mutation rate and, consequently, to the level of STR polymorphism. DNA repair mechanisms correct many replication errors, thereby reducing the frequency with which STR mutations become fixed in the genome. Studies comparing different STR motifs have shown that PCR-associated polymerase slippage and stutter formation occur more frequently in dinucleotide repeats than in tetranucleotide repeats [21]. Consequently, tetranucleotide markers are increasingly incorporated into newly developed forensic STR panels because they facilitate profile interpretation and reduce stutter artefacts. However, many routine STR panels currently used for animal parentage testing and individual identification still contain predominantly dinucleotide markers, reflecting their historical development and long-term validation [22,23,24]. Meredith et al. (2020) reported that tetranucleotide STR panels may provide a higher average number of alleles and improved analytical performance compared with dinucleotide repeat systems [21]. Variation in the number of repeat units among individuals, combined with relatively high mutation rates, contributes to the high level of polymorphism observed in STR loci [25,26,27]. In both human and animal forensic genetics, STR markers have largely replaced RFLP and VNTR analyses because of their high polymorphism, discriminatory power, and compatibility with PCR amplification from limited amounts of DNA [14].

## 4. Recent Developments in Animal STR Analysis

Recent years have brought substantial progress in animal forensic genetics, particularly in the field of short tandem repeat (STR) analysis. Traditional capillary electrophoresis (CE)-based methods, which differentiate alleles according to fragment length, are increasingly being complemented by massively parallel sequencing (MPS), also referred to as next-generation sequencing (NGS). Unlike conventional STR typing, sequence-based analysis enables the detection of isoalleles and sequence variation within alleles of identical length, including polymorphisms located in flanking regions. As a result, MPS significantly increases the discriminatory power of STR markers and facilitates the interpretation of complex DNA mixtures, kinship analyses, and population genetic studies [22,28].

The growing application of MPS technology has substantially influenced the development of animal forensic genetics. Modern multiplex STR systems developed for dogs and horses increasingly use tetra- and pentanucleotide repeats because of their lower stutter formation and improved interpretation reliability. Liu et al. (2024) developed a novel 30-plex canine STR assay for individual identification and parentage testing, while recent validation studies confirmed the usefulness of expanded canine STR panels in forensic investigations and breeding verification [22,23]. Similar advances have been achieved in equine genetics, where highly polymorphic tetra- and pentanucleotide markers enhance the accuracy of pedigree testing and population monitoring [29]. In addition, ongoing activities coordinated by the International Society for Animal Genetics (ISAG) contribute to the standardization and validation of STR marker panels used for parentage verification and individual identification across numerous domestic animal species [7].

Advances in STR analysis have also expanded its application beyond individual identification and parentage verification. STR markers are increasingly used in population genetic studies to assess genetic diversity, population structure, gene flow, and levels of inbreeding in both domestic and wild animal populations. Such applications provide valuable information for conservation genetics, breeding management, and the long-term monitoring of endangered species, complementing their established role in forensic investigations [24,30].

Wildlife forensic genetics has gained increasing attention owing to the growing scale of illegal wildlife trade and poaching. Current studies frequently combine STR markers with single-nucleotide polymorphisms (SNPs) in integrated SNPSTR systems, which provide greater discriminatory power and improved analysis of degraded samples. In 2025, the FOGS (Forensic Genetics for Species Protection) project introduced SNPSTR marker sets for 74 vertebrate species together with a dedicated forensic database supporting species identification, biodiversity conservation, and wildlife protection efforts. The database provides harmonized SNPSTR marker information that facilitates species identification and supports wildlife forensic investigations involving protected vertebrate species [31]. Despite their advantages, SNPSTR systems are associated with several analytical challenges. Common issues include amplification bias, allelic dropout in low-quality or degraded samples, sequencing errors, low-frequency false variants, and difficulties in bioinformatic processing and interpretation. These limitations may affect genotype calling and data reliability, particularly when analyzing mixed DNA samples or forensic traces from wildlife specimens. Therefore, robust validation procedures and standardized analytical workflows remain essential for the reliable implementation of SNPSTR markers in forensic and conservation genetics [28,30,31,32].

At the same time, the growing use of MPS-based sequence analysis of STR markers has highlighted the necessity for international standardization of nomenclature and interpretation procedures. Recent ISFG recommendations emphasize the need to maintain compatibility between traditional CE-based nomenclature and sequence-derived STR profiles generated by MPS technologies [32]. Recent studies have additionally shown that NGS-specific artefacts, including sequence noise and stutter variants, require updated interpretation algorithms and bioinformatic pipelines, particularly in low-template and mixed DNA samples [31].

## 5. Artefacts

Artefacts in genetic analysis can have a significant impact on the reliability of results. Stutter artefacts arise primarily from polymerase slippage during PCR amplification and become more problematic in low-template DNA samples and after extensive amplification [33]. Stutters are fragments differing in size from authentic alleles in one or more repeat units. These additional signal peaks can complicate data interpretation and require careful evaluation during genetic analysis. By the end of the 20th century, PCR amplification of dinucleotide STRs had already been observed to generate such artefacts, which posed a challenge for the analysis of these genetic markers. Although stutter artefacts occur in all STR types, they are generally less pronounced in tetra- and pentanucleotide markers than in dinucleotide repeats. Consequently, recently developed animal STR panels increasingly incorporate tetra- and pentanucleotide repeat motifs to improve profile interpretation and reduce stutter artefacts [6,21,34,35]. The International Society for Forensic Genetics (ISFG) recommends the use of tetranucleotide markers in non-human DNA analysis because of their lower levels of polymerase stuttering and heterozygote imbalance, which improve the reliability of genetic profile interpretation [10]. Larger repeat motifs are generally associated with lower levels of stutter formation, making allele assignment and profile interpretation more reliable [26]. Another important problem in analyzing a small amount of DNA is the occurrence of unfavourable phenomena such as stochastic effects. This is a situation in which an allele is not amplified (allelic drop-out), or an allele from an exogenous source is present (allelic drop-in). For this reason, it is crucial to establish a stochastic threshold, defined as a laboratory-specific RFU threshold used to support the interpretation of low-template DNA profiles and to reduce the risk of incorrect genotype interpretation resulting from stochastic effects [33,36].

During PCR, polymerase has a tendency to add one adenine base which is not present in the template strand. This results in a product with one additional base (n + 1 peak) [37]. To minimize interpretation difficulties associated with incomplete adenylation, PCR conditions are typically optimized to promote the consistent formation of adenylated products, thereby reducing the presence of both n and n + 1 peaks in the same electropherogram [33]. A product with one fewer base (n − 1 peak) can also occur [38]. In addition, preferential amplification may occur during PCR, resulting in the more efficient amplification of one allele at a heterozygous locus. This phenomenon may contribute to peak imbalance and complicate the interpretation of heterozygous genotypes, particularly in low-template or degraded DNA samples. However, unbalanced peaks should not be ignored, as they may also indicate chromosomal abnormalities, somatic mutations, or the presence of mixed DNA samples [33,36].

Another challenge in STR analysis is the occurrence of null alleles, which usually result from sequence variation within primer-binding sites that prevents amplification of one allele. Consequently, heterozygous individuals may be misclassified as homozygotes, potentially affecting parentage testing, population genetic analyses, and forensic interpretation. Careful marker validation and assay optimization are therefore essential to minimize their impact on STR analysis.

Additional artefacts associated with capillary electrophoresis include pull-up peaks resulting from spectral overlap between fluorescent dyes, which typically occur when very strong fluorescent signals, often caused by excessive DNA concentration, exceed the dynamic range of the detection system. Other artefacts include dye blobs caused by residual fluorescent molecules and off-scale peaks generated by detector saturation. Although these artefacts do not represent true genetic variation, they can complicate profile interpretation, particularly in mixed or low-template DNA samples [33,39].

Low-template DNA samples and DNA mixtures remain among the most challenging types of forensic evidence because they increase the risk of allelic dropout, peak imbalance, and profile interpretation errors [40]. When DNA is highly degraded, conventional STR analysis may become less effective because larger fragments often fail to amplify. In such cases, SNP markers may provide complementary information because they can be amplified using very short amplicons, often shorter than 100 base pairs, which improves the likelihood of obtaining genetic data from degraded samples [41].

The artefacts described above are inherent to DNA analysis and must be considered during result interpretation [39]. Several strategies have been proposed to reduce STR-associated artefacts. These include optimization of PCR conditions, reduction in amplification cycle numbers, the use of tetra- and pentanucleotide STR markers, and the application of modified or high-fidelity DNA polymerases designed to reduce polymerase slippage and stutter formation. In addition, sequence-based STR analysis may improve discrimination between true alleles and amplification artefacts [26,31,37].

## 6. Nomenclature

Traditionally, STR alleles in animal genetics have been designated according to amplicon length determined by capillary electrophoresis. When allele designation is based on amplicon length determined using a specific primer pair, subsequent analyses must employ the same primers to ensure comparability of results. The use of an alternative primer pair may alter the measured fragment length, even when the underlying STR sequence differs by only a single nucleotide [10,19]. Moreover, in the flanking regions of short tandem repeats, and thus at the sites of annealing of primers, indel mutations (insertions or deletions) can take place. In this case, when capillary electrophoresis (CE) is performed during the analyses, the allele peak corresponding to this fragment will be measured differently from the size of the allele ladder, even though the sequence of the region with repeats is the same [19]. Nomenclature based on repeat number rather than amplicon length facilitates data comparison among laboratories and improves compatibility between different analytical platforms. The increasing use of massively parallel sequencing (MPS) has further highlighted the importance of standardized allele nomenclature because sequence variation may occur within alleles of identical length. In human forensic genetics, recommendations for sequence-based STR nomenclature have been developed by the International Society for Forensic Genetics (ISFG) [32]. Similar standardization efforts are also undertaken in animal genetics through the activities of the International Society for Animal Genetics (ISAG), which supports harmonization of marker panels and reporting systems used for parentage verification and individual identification in numerous animal species [7]. Standardization is further supported through the harmonization of marker panels, allele nomenclature, and regular inter-laboratory comparison exercises, which improve the consistency and comparability of STR genotyping results between laboratories. Several recent studies have emphasized the need for broader implementation of sequence-based nomenclature systems in animal STR analysis to improve data exchange and inter-laboratory comparability [28,32]. An example of this approach was presented by Liu et al. (2024), who developed and validated a 30-plex canine STR assay and reported allele designations based on repeat number rather than fragment length [22].

## 7. The Use of Genetic Methods for Individual Identification

Genetic analyses are particularly important in the context of criminal proceedings, because they enable conclusive identification of the subject which is the source of a DNA trace [15]. Although forensic genetics initially focused on human DNA, biological material of animal origin has gradually become an important source of evidence in criminal investigations. Criminal cases in which non-human DNA has been analyzed have mainly concerned the theft of valuable animals, but have also included murder cases, in which the perpetrator was successfully linked to the site of the crime through biological material of animal origin that had been left there [5,40,42].

Dogs and cats were among the first animal species to attract attention in forensic genetic research because of their close association with humans. Considerable efforts have therefore been devoted to the development of genetic tools for their identification. Several STR-based systems have been developed for domestic cats, enabling reliable individual identification and parentage verification in forensic and breeding applications [8,43]. Similar developments have been achieved for dogs, for which dedicated multiplex STR panels are routinely used in forensic investigations and pedigree verification [23,44]. Applications of animal DNA profiling extend beyond forensic investigations and include parentage verification, breeding management, conservation genetics, wildlife forensic science, and population monitoring. STR markers are also routinely applied in livestock species for parentage verification, breeding management, traceability, and the maintenance of genetic diversity within breeding populations [45].

Beyond forensic investigations, animal STR analysis provides valuable information on genetic diversity within populations, supporting the management and conservation of both domestic and wild species. Horses represent an economically and culturally important domestic species, and the maintenance of genetic diversity within breeding populations is essential for sustainable breed management. Population genetic monitoring is particularly important because intensive selection for desirable traits may reduce allelic diversity and contribute to the loss of genetic variation [46]. STR panels for horses have also been developed, containing dinucleotide as well as tetra- and even pentanucleotide repeats, which are the most useful [24,46]. For the safety of people, a multiplex panel consisting of tetranucleotide STRs and markers identifying sex has been created for the purpose of individual identification of American black bears. This approach facilitates the identification of individual bears involved in human–wildlife conflicts and supports appropriate wildlife management decisions. Such analyses also help prevent unnecessary management actions against animals that were not involved in a given incident [21].

Considerable progress has also been made in the development of species-specific STR multiplex systems for endangered wildlife. Dedicated STR panels have been developed for several endangered species, including elephants, rhinoceroses, tigers, and lions, to support individual identification, parentage verification, population monitoring, and investigations of illegal wildlife trade. These genetic tools have become an important component of wildlife forensic science by facilitating the identification of confiscated specimens, tracing their geographic origin, and supporting conservation and law enforcement efforts [29,30,47].

Beyond species identification, genetic methods also play an important role in combating poaching, illegal wildlife trade, and trafficking of protected species [11].

Genetic analyses of endangered species present additional challenges. Many threatened populations are characterized by reduced genetic diversity and increased levels of inbreeding, which may decrease the discriminatory power of STR markers and complicate population genetic analyses [21,47]. Furthermore, numerous endangered species lack comprehensive reference databases, limiting the statistical interpretation of genetic evidence and the assignment of individuals to specific populations or geographic regions [30,47]. The collection, transport, and international exchange of biological samples are also subject to legal restrictions, particularly for species protected under CITES and other conservation regulations [30]. Despite these limitations, STR- and SNP-based approaches remain valuable tools for monitoring genetic diversity, identifying illegally traded specimens, assessing population structure, and supporting conservation management programmes [11,29].

Future progress in this field will depend both on the refinement of existing marker panels and on the identification of informative loci in species that remain poorly characterized genetically [35].

## 8. Current Limitations and Future Perspectives

Although standardized STR panels are available for several domestic animal species, many wildlife and non-model species still lack standardized marker panels, hindering comparison of results between laboratories and complicating data exchange among forensic and research institutions [32,47]. In many wildlife species, reference population databases are still incomplete or unavailable, limiting the statistical interpretation of forensic results and reducing the reliability of individual assignment and geographic origin analyses [32,48]. One such resource, the FOGS database, provides harmonized SNPSTR marker information for protected vertebrate species and facilitates species identification, population assignment, and wildlife forensic investigations [29]. Sequence-based STR analysis and massively parallel sequencing also generate large datasets that require standardized bioinformatic pipelines, quality assurance procedures, and harmonized nomenclature systems to ensure comparability of results across laboratories [32,48]. Future developments will likely focus on integrating STRs with SNP markers, expanding reference resources for wildlife forensic genetics, and implementing next-generation sequencing technologies in routine animal identification and conservation genetics [29,30,49].

## 9. Conclusions

STR analysis remains one of the most important methods used for genetic identification in both humans and animals. Owing to its high sensitivity, discriminatory power, and compatibility with multiplex PCR, it continues to play a central role in forensic investigations, biodiversity conservation, and parentage verification. Although challenges related to result interpretation and the development of species-specific marker panels persist, STR markers continue to provide a robust framework for animal genetic analyses. Future advances are expected to integrate STR analysis with complementary approaches, including SNP markers, massively parallel sequencing (MPS), and SNPSTR systems. Continued progress will rely on rigorous validation of newly developed marker panels, standardized nomenclature, advanced bioinformatic pipelines, and robust interpretation methods to further improve the accuracy and applicability of animal genetic analyses in forensic investigations, conservation genetics, and wildlife protection.

## Data Availability

No new data were created or analyzed in this study. Data sharing is not applicable to this article.
